# Identification and expression of CAMTA genes in *Populus trichocarpa* under biotic and abiotic stress

**DOI:** 10.1038/s41598-017-18219-8

**Published:** 2017-12-20

**Authors:** Ming Wei, Xuemei Xu, Chenghao Li

**Affiliations:** 1 State Key Laboratory of Tree Genetics and Breeding, Northeast Forestry University, 26 Hexing Road, Harbin, 150040 China; 2 Library of Northeast Forestry University, 26 Hexing Road, Harbin, 150040 China

## Abstract

The calmodulin-binding transcription activators (CAMTAs) transcription factor family plays an important role in normal plant growth and development, as well as in biotic and abiotic stress resistance. In this study, we identified seven *CAMTA* genes across the whole genome of *Populus trichocarpa* and analyzed the expression patterns of *PtCAMTAs* in the root and leaf tissues. Promoter *cis*-element analysis indicated that most *CAMTA* genes contained stress- or phytohormone-related *cis*-elements. Quantitative real-time reverse transcription-PCR (qRT-PCR) indicated indicated that *PtCAMTAs* were induced by mannitol, NaCl, cold stress, pathogenic infection with *A. alternata*, and phytohormone treatments with abscisic acid, salicylic acid, and methyl jasmonate. We analyzed the expression of homologous genes between *P*. *trichocarpa* and *P. ussuriensis* and alternative splicing forms of *PtCAMTA* genes under cold stress. We also performed a network interaction analysis for *PtCAMTA* proteins to predict their interactions and associations. The results of the present study serve as a basis for future functional studies on the *Populus CAMTA* family.

## Introduction

The divalent cation Ca^2+^, a universal secondary messenger in eukaryotic organisms, is employed by plants. Ca^2+^ signals are sensed through the actions of Ca^2+^-binding proteins, which contain the highly conserved Ca^2+^-binding ‘EF-hand’ motif that exhibits different configurations depending on the Ca^2+^ loading status^1^. In recent years, three classes of Ca^2+^ messenger, involving calmodulins and calmodulin-like proteins (CaMs and CMLs), calcium-dependent protein kinases (CDPKs), and calcineurin B-like proteins (CBLs) have been characterized in plants^2^. To date, it has been determined that CaMs can regulate at least 90 transcription factors, including calmodulin-binding transcription activators (CAMTAs)^3^.

CAMTAs, as a family of conserved transcription factors, have been found to exist in all multicellular eukaryotes and constitute the main transcription factors regulated by calmodulin. CAMTAs contain a specific CG-1 DNA-binding domain in the N terminus, followed by a TIG domain involved in non-specific DNA binding, an ankyrin (ANK) repeats domain, a Ca^2+^ dependent CaM binding domain (CaMBD) that is located between the N terminus and C terminus, and a different number of IQ motifs that interact with CaM in a Ca^2+^-independent pattern^4,5^. To date, several *CAMTA* genes from different plant species have been characterized, including *Arabidopsis, Medicago truncatula*, soybean, and maize^6–8^. The *Arabidopsis* CAMTA proteins group into four classes, namely, Ia, IIa, IIIc and IIIe, and the IIIe CAMTAs mostly constitute the non-TIG type with duplication occurring only in some species.

CAMTA transcription factors play an important role in plant growth and development, as well as in biotic and abiotic stress responses, particularly cold stress. Studies indicate that *AtCAMTA3* is a positive regulator of CBF2 expression, which is known to be involved in the cold stress response. *AtCAMTA1* and the *AtCAMTA3* double mutant were discovered to have low tolerance to freezing^9^. In addition, *AtCAMTA3* was found to possess 10 interactors, including CBF2. *AtCAMTA1* is also involved in the expression regulation of a broad spectrum of membrane integrity response genes via the generating of an ABA response to drought stress^10^. The CAMTA homologs were able to recognize the (A/C)CGTGT DNA motif, which encompasses a classic abscisic acid (ABA)-responsive element (ABRE, ACGTGT)^11,12^. Furthermore, the maize *ZmCAMTA1* was significantly reduced by cold treatment in the roots. The majority of *MtCAMTA* genes have been proven to respond to hormones such as SA, MeJA, and ABA, suggesting that CAMTA-mediated abiotic and biotic stress tolerance may exist in different plant species.

*Populus trichocarpa* is widely used in functional forest tree genomics studies and has significant commercial and ecological value^13^. *Populus trichocarpa* is threatened by a multitude of environmental stresses and biotic stresses such as drought and fungal disease during growth and development. Although *CAMTA* genes have been studied in some plants, research into the *CAMTA* gene family in *Populus* is limited. We identified seven *CAMTA* genes in *P*. *trichocarpa* and analyzed their phylogenetic relationships, chromosomal locations, gene duplication events, and gene structures. The expression mode of *PtCAMTAs* under abiotic stress (mannitol, NaCl, 4 °C), biotic stress (*Alternaria alternate* infection), and phytohormone treatment, including abscisic acid (ABA), salicylic acid (SA), and methyl jasmonate (MeJA), were analyzed using quantitative real-time RT-PCR (qRT-PCR). We also analyzed the expression of homologous *PtCAMTA* genes between *P*. *trichocarpa* and *P*. *ussuriensis* and alternative splicing forms of *PtCAMTA* genes under cold stress. The results may support further functional gene research through the study of these candidate *CAMTA* genes in response to abiotic and biotic stress.

## Results

### Identification, phylogenetic relationships, gene structure, conserved domain and alternative splicing analyses of PtCAMTA genes

We identified a total of seven *CAMTA* genes in *P*. *trichocarpa*, which encoded proteins that varied in length from 907 to 1,116 amino acids (aa), with an average length of 1,000 aa. The CAMTA protein sequences showed large variations in isoelectric point (pI) values (ranging from 5.45 to 8.27) and molecular weight (ranging from 102.195 kDa to 126.102 kDa). The location of the PtCAMTA proteins was predicted to be the cell nucleus using Wolf PSORT (Table 1).

**Table 1:** The *CAMTA* gene family in *Populus trichocarpa*.

**Gene name**	Transcript Name	Accession number	NCBI locus ID	Length (aa)	MW (Da)	pI	Localization
*PtCAMTA1*	Potri.001G057800.1	POPTR_0001s13700	XM_006368809.1	998	111418.7	5.45	nucl
*PtCAMTA2*	Potri.005G075100.1	POPTR_0005s07660	XM_002307047.2	1116	126102.3	5.52	nucl
*PtCAMTA3*	Potri.007G093400.1	POPTR_0007s05410	XM_002310526.2	1091	122444.9	5.49	nucl
*PtCAMTA4*	Potri.010G141700.1	POPTR_0010s15160	XM_002314890.2	915	102868.2	8.27	nucl
*PtCAMTA5*	Potri.010G153100.1	POPTR_0010s16290	XM_002316035.2	999	111276	7.14	nucl
*PtCAMTA6*	Potri.008G107900.1	POPTR_0008s10730	XM_002311358.2	907	102194.5	6.70	nucl
*PtCAMTA7*	Potri.003G170600.1	POPTR_0003s16910	XM_002303751.2	980	109542.2	5.53	nucl

**Figure 1:**
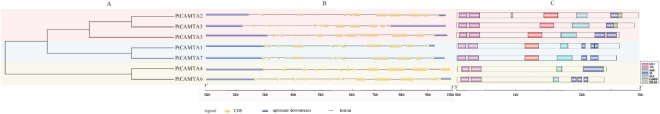
Phylogenetic relationships and gene structure of *Populus CAMTAs*. (**A**) Multiple alignment of full-length amino acid sequences of *Populus CAMTA* genes, executed in ClustalX 1.83. The phylogenetic tree was constructed using the neighbor-joining method in MEGA 5.0. Support values from a bootstrap analysis with 1,000 replicates are specified at each node. The three major phylogenetic subgroups are marked with different colored backgrounds. (**B**) Exon/intron structures. Exons and introns are represented by particular colored boxes and black lines, respectively. (**C**) Bioinformatics analysis of the conserved domains was conducted in the Pfam database (http://pfam.janelia.org/). Cam-binding domains (CaMBD) were specifically searched in the Calmodulin Target Database (http://calcium.uhnres.utoronto.ca/ctdb/ctdb/).

To examine the phylogenetic relationships among the CAMTA domain proteins in *P*. *trichocarpa*, an unrooted phylogenetic tree was constructed from the full-length *CAMTA* sequence alignments. We classified seven *CAMTA* genes into three subgroups according to their homology (Fig. 1A). A comparison of the exon/intron organization of the coding sequences of individual *PtCAMTA* genes showed a similar exon-intron structural pattern, indicating a necessary conservation of the genomic structure of *PtCAMTA* genes (Fig. 1B). The conserved domains of the CAMTAs, involving a CG-1 DNA binding domain, a TIG domain, ankyrin repeats, and one or two copies of IQ motifs were predicted in the PtCAMTA proteins (Fig. 1C).

We analyzed the *PtCAMTAs* primary cDNAs and the genomic DNA sequences. The results showed that *PtCAMTA1*-*7* produced splice variants. The number of unigenes corresponding to splice variants was two in *PtCAMTA1*, *4*, *6*, and *7*; three in *PtCAMTA3*; four in *PtCAMTA2* and five in *PtCAMTA5*. There are four major alternative splicing types of *PtCAMTAs*: intron retention (*PtCAMTA1.2*, *2.3*, *3.2*, *3.3*, *4.2*, *5.2* and *5.5*); alternative 5′ splice site (*PtCAMTA2.2*,*5.3*, *6.3*); alternative 3′ splice site (*PtCAMTA6.2*, *2.4*); and cassette exons (PtCAMTA7.2) (Supplementary Figure S1).

### Chromosomal location and duplication of PtCAMTA genes

To verify the relationship between genetic divergence and gene duplication, we identified the chromosomal locations of *PtCAMTA* genes. As shown in Supplementary Figure S2, *PtCAMTA* genes were characterized by an obvious feature whereby all the genes were distributed on chromosomes I, III, V, VII, VIII, and X, respectively. There were two *PtCAMTA* genes (*PtCAMTA3* and *PtCAMTA6*) on chromosome X. The rest of the *PtCAMTA* genes were detected on each of chromosomes I, III, V, VII, and VIII. In order to confirm the relationship between the *CAMTA* genes and potential segmental duplications, we used the duplicated blocks set up in a previous study. The distribution of the duplicate blocks related to *CAMTA* genes is illustrated in Supplementary Figure S2. We discovered that two of the seven *PtCAMTA* (*PtCAMTA1* and *PtCAMTA7*) genes were present in both duplicated regions and were thus prioritized, as the others were only present in one of the blocks. The history of selection acting on coding sequences can be measured on the basis of the ratio of nonsynonymous to synonymous substitutions (Ka/Ks). A pair of homologous sequences will have Ka/Ks < 1 if one sequence has been under the select of purification but the other has been drifting neutrally, while Ka/Ks = 1 when both sequences are drifting neutrally and peculiarly, and Ka/Ks > 1 at specific sites that are under positive selection^14^. A summary of Ka/Ks for three CAMTA duplicated pairs shown in Supplementary Table S1 was less than 0.7. This result suggests that all gene pairs have evolved mainly under the influence of purifying selection. Based on the divergence rate of 6.1 × 10^−9^ synonymous mutations per synonymous site per year as previously presented for populus^15^, duplications of these three paralogous pair genes was estimated to have taken place between14.7 to 147.4 Mya (Supplementary Table S1).

### Promoter cis-element analysis

**Figure 2:**
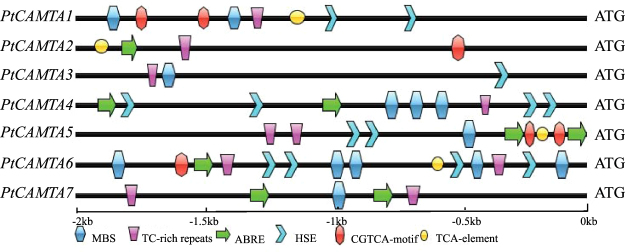
Abiotic stress and phytohormone response elements in *PtCAMTA* genes promoter.

We identified putative *cis*-acting regulatory DNA elements via the promoter sequences of *PtCAMTA* genes (2,000 bp upstream of the translation start site) based on the Phytozome version 12.1 database. The *CAMTA* gene family promoter sequences demonstrated that several *cis*-elements were related to biotic and abiotic stress responsiveness (Fig. 2). In total, 10 types of abiotic stress elements were identified. Nearly all the *PtCAMTA* genes had MBS elements and five had W-box in their promoters, which showed that the MYB binding site is involved in drought inducibility. Three of the *PtCAMTA* genes (*PtCAMTA4*, *PtCAMTA5*, and *PtCAMTA6*) possessed AREB-responsive elements (ABREs). Nearly all the *PtCAMTA* genes possessed MeJA-responsive elements (CGTCA-motif, G-Box, TGACG-motif), except *PtCAMTA6*. Four of the *PtCAMTA* genes (*PtCAMTA1*, *PtCAMTA3*, *PtCAMTA5*, and *PtCAMTA6*) possessed SA-responsive elements (TCA-element; Supplementary Table S2).

### Tissue-specific expression profile

We observed specific expression patterns in the different tissues of *PtCAMTA* genes from the Affymetrix (GSE6422) microarray data in PopGeneIE version 3.0 (Supplementary Figure S3). The microarray data showed high expression levels of *PtCAMTA* genes in the roots, with *PtCAMTA3* exhibiting particularly high expression levels among all the *PtCAMTA* genes. In the leaves, only *PtCAMTA1* was highly expressed in the mature leaves. We found that *PtCAMTA2* and *PtCAMTA3* displayed low expression levels in the mature leaves and high expression levels in the roots, simultaneously. Notably, the expression level of all *PtCAMTAs* in the young leaves was very low.

### Expression analysis of PtCAMTA genes under mannitol and NaCl stress

**Figure 3:**
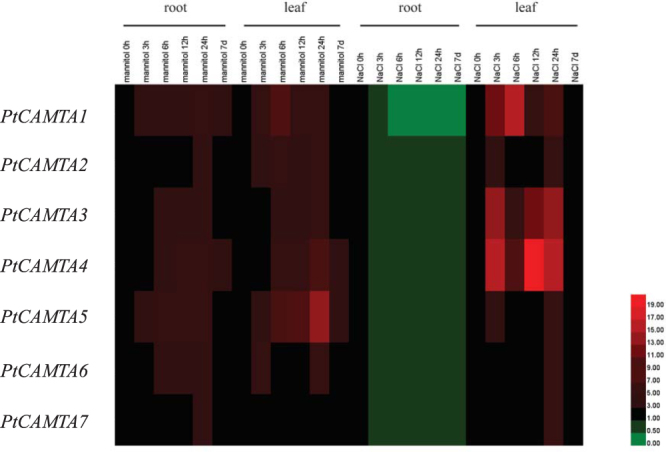
Expression analysis of *PtCAMTA* genes in the roots and leaves under mannitol and NaCl stress by qRT-PCR. Red and green indicate high and low levels of transcript abundances, respectively.

In order to understand how *PtCAMTA* genes react under osmotic stress, we analyzed the expression of the *PtCAMTAs* in the roots and leaves under treatment with 200 mM mannitol and 150 mM NaCl for 3 h, 6 h, 12 h, 24 h, and 7d, respectively. Genes that were up or downregulated by more than 2.0-fold were considered significantly differentially expressed^16^. Under mannitol stress, all the genes were upregulated in both the roots and leaves in the short-term treatments (3 h, 6 h, 12 h, and 24 h). Changes in the expression of *PtCAMTA3*, *6*, and *7* were not obvious in the roots and leaves in the long term (7 d). Notably, *PtCAMTA1*, *4*, and *5* were significantly upregulated (>5.0-fold relative to the control) in the roots. *PtCAMTA1*-*6* was significantly upregulated in the leaves. Under NaCl stress, all the *PtCAMTA* genes were suppressed in the roots at all time points. *PtCAMTA* genes were upregulated in the leaves under short-term stress and showed no change under long-term stress (Fig. 3).

### The expression of PtCAMTA genes induced by pathogenic infection with ***A. alternata***

**Figure 4:**
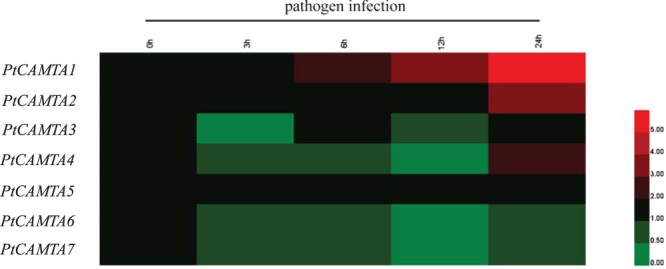
Expression analysis of *PtCAMTA* genes in leaves under pathogen infection by qRT-PCR. Red and green indicate high and low levels of transcript abundances, respectively.

Poplar leaf blight, caused by the pathogen *A. alternata*, is a common disease in northeast China. In order to study the expression of *PtCAMTA* genes under biotic stress, we infected the leaves of *P*. *trichocarpa* with *A. alternata in vitro*. Following infection, two *CAMTA* genes (*PtCAMTA1* and *2*) were induced, four *CAMTA* genes (*PtCAMTA3*, *4*, *6*, and *7*) were suppressed, and *PtCAMTA5* showed no change. *PtCAMTA1*, *2*, and *4* were induced at 24 h after infection (Fig. 4).

### Expression levels of PtCAMTA genes in response to phytohormone stimuli

**Figure 5:**
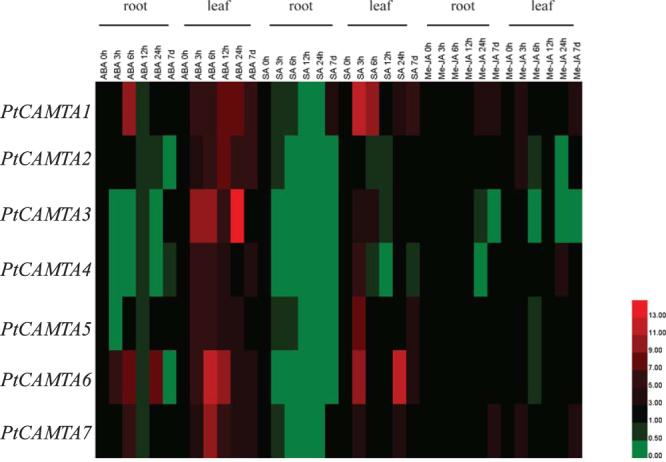
Expression analysis of *PtCAMTA* genes in the roots and leaves under ABA, SA, and MeJA treatments by qRT-PCR. Red and green indicate high and low levels of transcript abundances, respectively.

To understand how *PtCAMTA* genes participate in stress-related hormone responses, we analyzed the expression of *PtCAMTA* genes under 200 μM ABA, 100 μM SA, and 100 μM MeJA in the leaves and roots for 3 h, 6 h, 12 h, 24 h, and 7d using qRT-PCR. Under ABA stress, three genes were upregulated before 6 h stress, and four genes were downregulated in the roots, while in the leaves all the genes were upregulated at all time points. Five *CAMTA* genes (*PtCAMTA1*, *2*, *3*, *6*, and *7*) were significantly upregulated in the short term. Under SA stress, all genes were significantly downregulated in the roots. Four genes (*PtCAMTA1*, *5*, *6*, and *7*) were upregulated in the leaves, whereas *PtCAMTA2*, *3*, and *4* were downregulated. Under MeJA stress, four genes (*PtCAMTA1*, *5*, *6*, and *7*) were upregulated, two (*PtCAMTA3* and *4*) were downregulated, and *PtCAMTA2* exhibited no change in the roots. With respect to the leaves, three genes (*PtCAMTA1*, *4*, and *7*) were upregulated and three (*PtCAMTA2*, *3*, and *5)* were downregulated. *PtCAMTA1* and *7* were induced in both the roots and leaves. Most *CAMTA* genes were downregulated in the roots under ABA, SA, and MeJA treatments (Fig. 5).

### Expression analysis of PtCAMTA alternative splicing forms under cold stress

Alternative splicing, as a post-transcriptional mechanism of precursor-mRNA, plays a significant role in transcriptome and proteome diversity as well as transcript and protein abundance^17,18^. As the *CAMTA* gene family is mainly involved in the cold stress response, we studied the expression of several alternative splicing forms of *PtCAMTA* genes under cold stress in detail.

**Figure 6:**
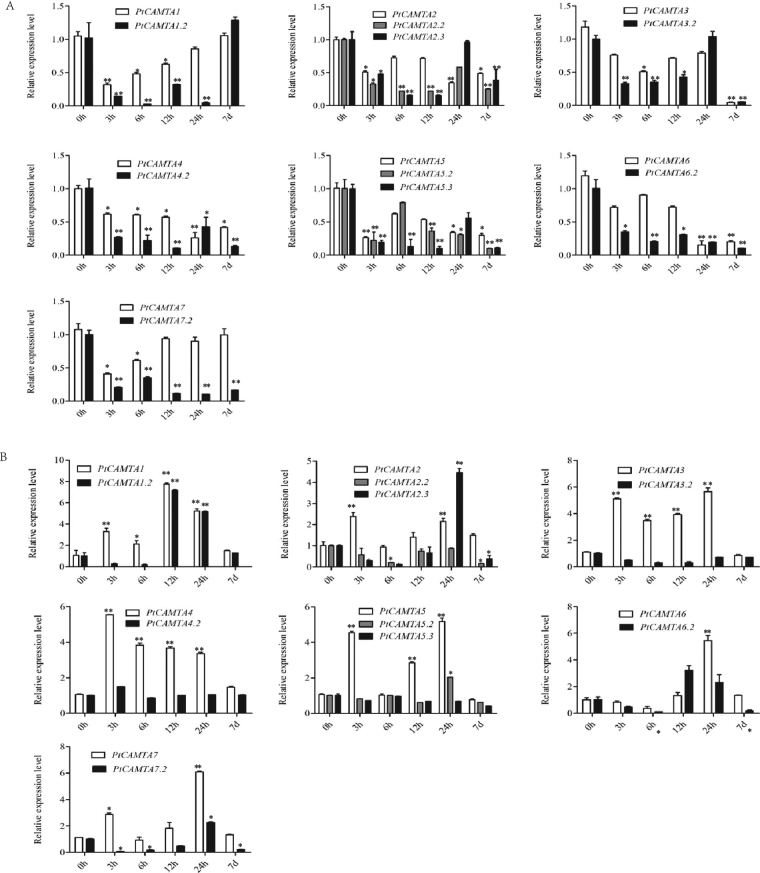
Expression analysis of alternative splicing forms of *PtCAMTA* genes under 4 °C stress. (**A**) expression analysis of alternative splicing forms of *PtCAMTA* genes in the roots under 4 °C stress. (**B**) expression analysis of alternative splicing forms of *PtCAMTA* genes in the leaves under 4 °C stress. The x-axis represents time after the onset of stress treatments. Error bars represent the standard deviations of three biological replicates. Asterisks indicate stress treatment groups that showed a significant difference in transcript abundance compared with the control group (*P < 0.05, **P < 0.01).

The analysis of qRT-PCR using splice variant-specific primers showed that specific amplicons could be obtained for *PtCAMTA1.2*, *PtCAMTA2.2*, *2.3*, *PtCAMTA3.2*, *PtCAMTA4.2*, *PtCAMTA5.2*, *5.3*, *PtCAMTA6.2*, and *PtCAMTA7.2*. Under cold treatment, most of the genes were downregulated in the roots in the short term, with the exception of *PtCAMTA1* and *PtCAMTA7*. At 7 d, the expression of *PtCAMTA2*-*6* in the roots was distinctly downregulated. The expression patterns of the splice variants in the roots were similar to the normal transcripts under cold treatment. Most of the splice variants of *PtCAMTAs* were downregulated in the roots under cold treatment. In the leaves, most of the genes were induced under cold treatment in the short time. *PtCAMTA1.2*, *2.3*, and *6.2* were induced in the leaves in the short time. The expression of *PtCAMTAs* in the leaves showed no significant change in the long term, but all the splice variants of *PtCAMTAs* in the leaves exhibited a negative trend at the same condition. In conjunction, these results suggest that *PtCAMTA*s are important in cold-regulated gene expression (Fig. 6).

### Homologous PtCAMTA genes in two Populus varieties under cold stress

**Figure 7:**
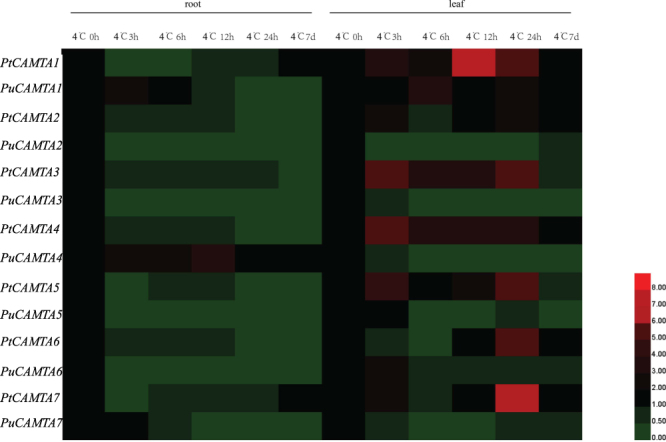
Expression analysis of *CAMTA* genes in the roots and leaves in *P*. *trichocarpa* and *P*. *ussuriensis* under 4 °C stress. Red and green indicate high and low levels of transcript abundances, respectively.

In order to gain further insight into the relationship between *PtCAMTA* gene expression and cold stress resistance, we compared the expression patterns of *P*. *trichocarpa* and *P*. *ussuriensis* (*Sect*. *Tacamahaca*: the same as *P*. *trichocarpa*. *P*. *ussuriensis* Kom), which are mainly distributed in the cold temperature zone of Northeast China and the far east region of Russia. *Populus trichocarpa* is a cold-tolerant species that can survive an annual average temperature of −3.8 °C as well as an extremely low temperature environment (−46.9 °C). Exhibiting strong resistance to cold, it constitutes the ideal candidate for studying the molecular mechanisms of woody plants. The results of the qRT-PCR revealed that the *CAMTAs* were differentially expressed in *P*. *trichocarpa* and *P*. *ussuriensis* under cold stress. For *P*. *ussuriensis*, the expression of most *PuCAMTA* genes was downregulated in the roots (except *PuCAMTA4*) and the leaves (except *PuCAMTA1*). The same trends were observed in *P*. *trichocarpa*, in that most of the *PtCAMTAs* were downregulated in the roots under cold treatment. However, most of the *PtCAMTA* genes were upregulated in the leaves in *P*. *trichocarpa* in the short term (Fig. 7).

### Protein interaction network analysis

**Figure 8:**
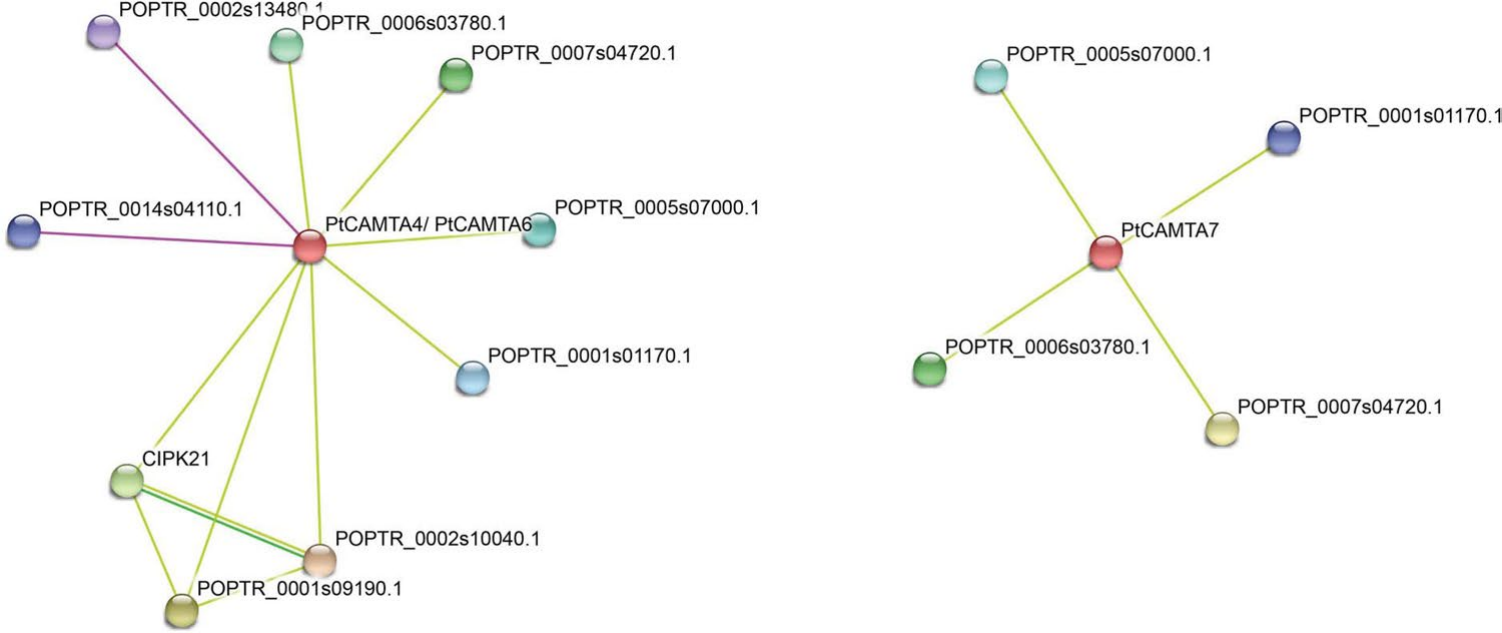
Protein interaction network of PtCAMTA proteins. The potential interactors of PtCAMTA4/6 and PtCAMTA7 were predicted using STRING software. The various interactions are illustrated with different colored connective lines.

In order to predict the interactions and associations of all the PtCAMTA proteins, we performed a network interaction analysis based on *Arabidopsis* proteins using STRING software, with the confidence value set at 0.5. An *Arabidopsis* CAMTA proteins network was created and 38 interactive proteins (confidence value = 0.5) were identified with the STRING database^19^. Then, the homologs of these 38 proteins in *Populus* were identified using Phytozome version 12.1. A total of eight unique proteins were predicted as potential interactors of PtCAMTA4, 6, and 7. The partners of PtCAMTA4 and 6 were predicted to be the Ca^2+^/CaM-regulated protein kinases CIPK5 and CIPK21. Moreover, PtCAMTA4 and PtCAMTA6 were identified as homologous proteins, which were predicted by STRING to directly interact or function in the same pathway (Fig. 8).

Studies have shown that CAMTA regulates the expression of the target gene by directly binding to the CGCG cis-elements of their promoter^20^. Rahman *et al*. have reported AtCAMTA3 protein can interacte with 10 proteins which contain at least one CGCG *cis*-element in their promoters in *Arabidopsis* ^21^. We found 16 homologous genes of the 10 *AtCAMTA3* target genes in *P. trichocarpa* using the Phytozome version 12.1 database, six of which were homologous genes. However, we discovered that only four homologous genes possessed CGCG *cis*-elements in their promoters (2,000 bp upstream of the translation start site) of the *AtCAMTA3* target genes in *P*. *trichocarpa*. (Supplementary Table S3)

## Discussion

In this report, seven *P*. *trichocarpa CAMTA* family gene members were identified, each of which contained conserved domains related to CAMTA proteins. Previous studies have suggested that the *Populus* genome experienced at least three rounds of genome-wide duplication, including multiple segmental duplication, tandem duplication, and transposition events such as retroposition and replicative transposition. Tandem and segmental duplication plays an important role in genomic expansions and realignments^22,23^. In our study, all the *PtCAMTA* genes were located in the duplicated regions. We discovered that *PtCAMTA1* and *PtCAMTA7* belonged to the same branch of the phylogenetic tree and were present in the homologous regions of chromosomes I and III, respectively. This finding might be explained by the homology of the two genes. As found in the CAMTAs that have been characterized in other species, all seven of the *PtCAMTAs* contained conserved domains. In one of the subgroups that contained CaMBD, we identified variation in *PtCAMTA2* and *PtCAMTA3* that was not present in the other genes. Based on the phylogenetic tree, we discovered that a close relationship exists between *PtCAMTA2* and *PtCAMTA3*. Interestingly, *PtCAMTA2* and 3 were found to be closely associated with *AtCAMTA3*. These have been well studied and are known to participate together in SA-mediated defense responses and cold tolerance^24^. These results suggest that *CAMTA* genes have divergent functions in *P. trichocarpa*.

Promoter *cis-*elements play crucial roles in the response to biotic and abiotic stresses^25^. In this study, we identified many abiotic stress responsiveness *cis*-elements in the promoters of *PtCAMTA* family genes, including MBS, ABRE, TCA-element, G-Box, and W-Box. In particular, *PtCAMTA5* was found to possess seven abiotic stresses responsiveness *cis*-elements, suggesting important functions under abiotic stress. Interestingly, *PtCAMTA1*, *2*, and *3* did not contain ABRE elements in their −2kb promoters, but their responsiveness to ABA treatment was clear. We can thus speculate that it is inaccurate to assume a correlation between the existence and responsiveness to the related stress treatments. The same conditions were found in soybean, in that *GmCAMTA7* and *GmCAMTA9* contained ABRE elements in their promoters, but their responsiveness to ABA treatment was not obvious^7^. In particular, most *PtCAMTA* genes have SA and MeJA related *cis*-elements (TCA-element, and G-Box, CGTCA-motif, and TGACG-motif), but no fungus related *cis*-element. The qRT-PCR results showed that *PtCAMTA2* and *PtCAMTA3* were induced not only by SA and MeJA, but also by infection with *A. alternata*. This result suggests that a close relationship exists between SA and MeJA with regards to biotic stress.

Plants are often affected by abiotic and biotic stress in the process of growth and development. Under mannitol and NaCl stress, all the *PtCAMTAs* were induced in the leaves. Conversely, all the *PtCAMTA* genes were downregulated in the roots under NaCl stress. We suggested that all the *PtCAMTA* genes have significant functions under mannitol and NaCl stress. Previous studies have shown that all the *GmCAMTA* genes and the majority of *ZmCAMTA* genes are induced in the roots under drought and NaCl conditions, indicating that different expression patterns exist between woody plants and crops^8^. *Alternaria alternata* is a fungus that has been recorded to cause leaf spot and other diseases on over 380 host species of plant^26^. In the present study, all *PtCAMTA* genes were differentially expressed in the leaves under *A. alternata* infection. Three *PtCAMTA* genes were upregulated under the 24 h infection period, while four were downregulated. A previous study showed that the expression of all but one of the *MtCAMTA* genes was significantly downregulated under biotic stress (*Sinorhizobium meliloti* infection)^6^. These results indicate different expression patterns of *CAMTA* genes under biotic stress across a variety of species. In particular, four *PtCAMTA* genes that were downregulated under *A. alternata* infection exhibited similar expression patterns under SA and MeJA stress. This result indicates that *PtCAMTA* genes are involved in the pathogen expression network related to SA and MeJA pathways.

Research has indicated that a variety of hormone responses to biotic and abiotic stress could mediate the expression of *CAMTA* in *Arabidopsis*, including ABA (*AtCAMTA2*, 4, *5*, and 6), SA (*AtCAMTA2*, 4, *5*, and 6), and MeJA (*AtCAMTA1*, *3*, and *4*)^27^. It has been reported that ABA is involved in the mediation of drought stress, and that it is an important regulatory factor during drought stress^28^. Research has also demonstrated that SA and MeJA constitute the two major plant hormones that regulate plant biotic stress signal transduction^29^. In our study, most of the *PtCAMTA* genes were differentially expressed in the roots and leaves under ABA, MeJA, and SA treatments. This result indicates that phytohormones regulate the expression of *PtCAMTA* genes. *PtCAMTA2* and *PtCAMTA3*, as homologous genes, were downregulated in the roots under ABA, MeJA, and SA treatment. Conversely, two soybean homolog genes (*Glyma05g31190* and *Glyma08g14370*) in *PtCAMTA2* and *PtCAMTA3* were induced in the roots under ABA, SA, and MeJA treatment^7^. These variations indicate that different expression patterns of *CAMTA* genes under abiotic stress and phytohormone treatment exist between woody plants and crops.

Cold stress is a major environmental factor that can affect plant growth and development. Previous studies have shown that CAMTA transcription factors play an important role in cold regulation. *AtCAMTA* genes have been reported to be involved in cold stress, and the *AtCAMTA1* and *AtCAMTA3* double mutant in particular was found to have a negative effect on freezing tolerance^30^. Previous studies have shown that *AtCAMTA* genes might regulate freezing tolerance via the SA signaling pathway. *AtCAMTA3* was found to be a negative regulator of the SA signaling pathway, and elevated levels of endogenous SA can enhance plant defense responses^31^. A study has shown that *AtCAMTA3* is induced under cold stress, while *AtCAMTA1* and *AtCAMTA*2 are downregulated under cold stress. Conversely, *PtCAMTA2* and *PtCAMTA3*, the homologs of *AtCAMTA1, 2* and *3*, were reduced in *P*. *trichocarpa*. According to the expression of several alternative splicing forms of *PtCAMTA* genes, we found that most of the splice variants of the *PtCAMTAs* were also downregulated in the roots, but induced in the leaves under cold stress. Alternative splicing plays a key regulatory role in modulating gene expression during development, and in response to environmental stimuli^32^. In this study, *PtCAMTA4.2* was more clearly expressed in leaves under cold stress. However, the expression of *PtCAMTA4* was not significant. These might be related to the absence motifs of *PtCAMTA4.2* alternative splicing variants which shown in Supplementary Figure S4. In conjunction, we believe these results indicate that *PtCAMTA* genes play an important role in the cold stress response, and that there are differences between *P*. *trichocarpa* and *Arabidopsis* in the response of *CAMTA* to cold stress. Furthermore, the expression patterns of *CAMTA* in *P*. *trichocarpa* differ from that of *P*. *ussuriensis*, in that most of the genes were clearly downregulated in the leaves under cold stress. We suggest that the differences in expression of the *CAMTA* genes under cold stress may be closely related to the differential cold tolerance of *P. trichocarpa* and *P. ussuriensis*.

In this study, we predicted eight potential *PtCAMTA* interactors using STRING software. The most consistent and interesting finding of this analysis was the different protein interactions between *PtCAMTAs* and *AtCAMTAs*. *PtCAMTA2* and *PtCAMTA3* share a high degree of homology with *AtCAMTA3*, which is known to be involved in both plant disease resistance and abiotic stress responses^33,34^. Rahman, H. *et al*. reported that *AtCAMTA3* possessed 10 interactors that are DNA-binding transcription factors, including SRS, CBP60G, CM2, ICE1, XLG2, RHL41/ZAT12, CBF1, CBF2, EDS1, and EDS16/ICS1. However, no protein that interacts with PtCAMTA2 and PtCAMTA3 was predicted using STRING software in the present study. This might be related to the absence of the CGCG CAMTA-binding element in the promoter of the homologous genes of the *AtCAMTA*3 interactors in *P*. *trichocarpa*. This result indicates that the regulatory pathway of PtCAMTA proteins is not the same as AtCAMTA proteins. An earlier study showed that the 38 potential interactors of *AtCAMTAs* are related to Ca^2+^ signaling components, such as Ca^2+^/CaM-regulated protein kinases, Ca^2+^-dependent phospholipids, and CaM-binding proteins^35^. According to the protein interactions analyzed in this study, none were related to Ca^2+^ signaling components among the eight potential interactors of PtCAMTA proteins. We boldly speculate that other binding sites exist besides the CGCG CAMTA-binding element in the promoter of the homologous genes of the *AtCAMTA*3 interactors in *P*. *trichocarpa* that act with PtCAMTA proteins. Another speculation is that *PtCAMTA* proteins act with the other DNA binding transcription factors to regulate Ca^2+^-related biological processes. The initial results of the present study have provided essential information on the *Populus* CAMTA family that may serve as basis for future functional studies, and will facilitate future mechanistic research aiming to investigate the divergent roles of these genes.

## Materials and Methods

### Identification of PtCAMTA genes

*Populus trichocarpa* genome sequence data were downloaded from the Phytozome version 12.1 (http://phytozome.jgi.doe.gov/pz/portal.html) and NCBI (http://www.ncbi.nlm.nih.gov/) databases. WoLFPSORT (http://wolfpsort.org/)^36^ was used to predict the subcellular localization of the CAMTA proteins. The ExPasy site (http://web.expasy.org/protparam/)^37^ was used to calculate the molecular weight and isoelectric point (pI) of the deduced polypeptides. Multiple alignment of the *PtCAMTA* full-length protein sequences was performed using ClustalX (version 1.83) and aligned manually using BioEdit 7.1 software^38^.

### Gene structure, chromosome localization, and gene duplications

The coding domain sequences (CDS) and DNA sequences of the *P. trichocarpa CAMTA* genes were used to reveal the gene structure using the Gene Structure Display Server (http://gsds.cbi.pku.edu.cn/index.php)^39^. The Multiple Expectation Maximization for Motif Elucidation (MEME) system (Version 4.9.1, http://meme.nbcr.net/meme/) was used to identify conserved motifs for each CAMTA gene^40^. The Softberry (http://linux1.softberry.com/berry.phtml?topic=fgenesh&group=programs&subgroup=gfind) was used to generate the exon/intron organization. Bioinformatics analysis of the conserved domains was conducted using the Pfam database (http://pfam.janelia.org/). The domain structures of the *PtCAMTAs* were drawn using Domain Illustrator software (http://dog.biocuckoo.org/)^41^. In order to confirm the chromosomal locations of the *CAMTA* genes, all *PtCAMTA* genes were obtained from the PopGenIE version 3 database (http://www.popgenie.org/). The *PtCAMTA* genes defined as separate by five or fewer gene loci within a genetic distance of 100 kb were considered to be tandem duplicates^42^. A schematic view of the reorganization of homologous chromosome segments was based on the most recent account of whole-genome duplication in *P. trichocarpa* ^43^.

### Calculation of Ka/Ks values

Pairs from the homologous genes were aligned by MEGA5.0. Subsequently, the aligned sequences were analyzed by the DnaSP program to calculated Ks and Ka rates. For each gene pair, the Ks value was translated into divergence time in millions of years based on a rate of 6.1 × 10^−9^ substitutions per site per year. The divergence time (T) was calculated as T = Ks/2 × 6.1 × 10^−9^ Mya.

### Promoter *cis*-element analysis

Promoter sequences (2 kb upstream of the translation start site) of all *CAMTA* genes were obtained from the Phytozome version 12.1 database. PlantCARE (http://bioinformatics.psb.ugent.be/webtools/plantcare/html/)^44^ was used to analyze the sequences of the *CAMTA* gene promoters and to predict and locate their cis-elements.

### Plant materials, abiotic stress, and phytohormone treatments

Clonally propagated *P*. *trichocarpa* (genotype Nisqually-1) and *Populus ussuriensis* (originated from seeds collected from 20-year-old trees growing at Liangshui Forest Farm of Northeast Forestry University, Yichun city, Heilongjiang province, China) were grow in half-strength Murashige and Skoog medium (1/2 MS) under long day conditions (16 h light, 8 h dark) at 25°C. *P*. *trichocarpa* plants were exposed to the following: 200 mM mannitol, 150 mM NaCl, 4 °C, 200 μM abscisic acid (ABA), 100 μM salicylic acid (SA), and 100 μM methyl jasmonate (MeJA). Each treatment lasted for 0 h, 3 h, 6 h, 12 h, 24 h, and 7 d, and samples were collected at each time point. Each experiment was repeated at least three times. Non-treated plants were used as controls. Additionally, *P*. *ussuriensis* plants were treated under a 4 °C stress treatment, as was *P*. *trichocarpa*. Following sampling, all samples were immediately frozen in liquid nitrogen and stored at −80 °C until analysis.

### *In vitro* pathogen inoculation assay

To induce fungal infection, mycelial plugs of the Populus leaf blight pathogen *Alternaria alternata* were placed on excised leaves and cultured in 1/2 MS medium^45^. The leaves were collected at 0 h, 3 h, 6 h, 12 h, and 24 h following fungal treatment. Three independent biological replicates were performed for each treatment. All collected samples were instantly frozen and stored at −80 °C for RNA isolation.

### RNA extraction and qRT-PCR analysis

Total RNA was extracted using the CTAB method^46^. cDNA was obtained using the PrimeScript™ RT reagent kit (Perfect Real Time; Takara, Dalian, China). SYBR Premix Ex Taq II (TaKaRa, Dalian, China) was used to perform qRT-PCR in 96-well optical reaction plates (Applied Biosystems, Foster City, CA, USA). Reactions were prepared in 20 μL volumes containing the following: 10 μL of 2× SYBR Premix, 6 μL of ddH_2_O, 2 μL of template, and 1 μL of each specific primer, prepared to a final concentration of 10 µM. In order to ensure the accuracy of the results, we performed three technical replicates for each sample. All primers mentioned above are listed in Supplementary Tables S4 and S5.

### Protein interaction network analysis

The online database resource search tool STRING 10 (http://string-db.org/) was used to predict the *PtCAMTA* protein interaction network. The STRING database integrated information from different datasets, including test and manually curated gene neighborhoods, gene fusion, gene coexistence, protein-protein interactions, and co-expressed genes to calculate the statistics and semantic links between proteins^47^.

### Accession numbers

*P*. *trichocarpa* genome sequence data (*PtCAMTA1*-*7*) were downloaded from the Phytozome version 12.1 (http://phytozome.jgi.doe.gov/pz/portal.html) and NCBI (http://www.ncbi.nlm.nih.gov/) databases with the following accession number: POPTR_0001s137001, POPTR_0007s054102, POPTR_0005s076601, POPTR_0010s151601, POPTR_0010s162901, POPTR_0008s107302, POPTR_0003s169101. *P*. *ussuriensis* sequence data (*PuCAMTA1*-7) can be found at NCBI with the following number: MF372148-54.

## Electronic supplementary material

Supplementary Information
