# Supplementary material for: Identification and expression of CAMTA genes in Populus trichocarpa under biotic and abiotic stress
Source: Sci Rep. 2017 Dec 20;7:17910. doi: 10.1038/s41598-017-18219-8 (PMC5738416; doi:10.1038/s41598-017-18219-8)
Supplement: Supplementary file 1 — Supplementary Information [file 41598_2017_18219_MOESM1_ESM.doc]

**Identification and expression of CAMTA genes in *Populus trichocarpa* under biotic and abiotic stress**

Ming Wei^1^, Xuemei Xu^2^, Chenghao Li^1*^

^1^State Key Laboratory of Tree Genetics and Breeding, Northeast Forestry University, 26 Hexing Road, Harbin 150040, China (e-mail: weiming920312@163.com);

^2^Library of Northeast Forestry University, 26 Hexing Road, Harbin 150040, China (e-mail: chli0@daum.net);

^*^Corresponding author (e-mail: chli0@163.com).

**Supporting Information**

**Supplementary Figure S1. Extons and introns of *PtCAMTAs* alternative splcing variants.**

Grey boxes represent 5’ UTR and 3’UTR, black boxes indicate exons, and black lines denote introns. The numbers indicate the size of each UTR, exon and intron (bp). The cut lines represent the missing segments.

**Supplementary Figure S2. Chromosomal locations of *PtCAMTA* genes.**

Segmental duplicated homologous blocks are indicated by the same color. The scale represents mega bases (Mb). The LG numbers are indicated above each bar.

**Supplementary Figure S3. Organ-specific expression of *PtCAMTA* genes in mature-leaves, young-leaves, roots, nodes, and internodes.** Each gene is indicated in every graph.

**Supplementary Figure S4. motifs of *PtCAMTAs* alternative splcing variants.**

**Supplementary Figure S5. Multiple sequence alignment of *PtCAMTA*1 alternative splicing.**

The black box represents the primer sequence

**Supplementary Figure S6. Multiple sequence alignment of *PtCAMTA*2 alternative splicing.**

The black box represents the primer sequence

**Supplementary Figure S7. Multiple sequence alignment of *PtCAMTA*3 alternative splicing.**

The black box represents the primer sequence

**Supplementary Figure S8. Multiple sequence alignment of *PtCAMTA*4 alternative splicing.**

The black box represents the primer sequence

**Supplementary Figure S9. Multiple sequence alignment of *PtCAMTA*5 alternative splicing.**

The black box represents the primer sequence

**Supplementary Figure S10. Multiple sequence alignment of *PtCAMTA*6 alternative splicing.**

The black box represents the primer sequence

**Supplementary Figure S11. Multiple sequence alignment of *PtCAMTA*7 alternative splicing.**

The black box represents the primer sequence

**Supplementary Table S1. Divergence time between *PtCAMTA* genes**

| **Paralogous pairs** | **Ks** | **Ka** | **Ka/Ks** | **Divergent date (Mya)** |
| --- | --- | --- | --- | --- |
| *PtCAMTA1-PtCAMTA7* | 0.1789 | 0.0813 | 0.4544 | 14.7000 |
| *PtCAMTA2-PtCAMTA3* | 0.2339 | 0.0754 | 0.3224 | 19.1000 |
| *PtCAMTA4-PtCAMTA6* | 1.7993 | 1.3001 | 0.7226 | 147.4000 |

**Supplementary Table S2. Abiotic stress and phytohormone related *cis***-elements.

| **Element** | **Core sequence** | **Function annotation** |
| --- | --- | --- |
| ABRE | TACGGTC | Response to abscisic acid |
| CGTCA-motif | CGTCA | Response to MeJA |
| ERE | ATTTCAAA | Response to ethylene |
| G-Box | CACGTT | Response to MeJA |
| HSE | AAAAAATTTC | Response to heat stress |
| MBS | TAACTG | MYB binding site involved in drought-inducibility |
| TCA-element | GAGAAGAATA | Response to salicylic acid |
| TGACG-motif | TGACG | Response to MeJA |
| W-Box | TTGACC | WRKY binding site involved in abiotic stress responsiveness |
| TC-rich repeats | GTTTTCTTAC | Response to defense and stress |

**Supplementary Table S3. 16 homologous genes of the *AtCAMTA*3 target genes in *P*. *trichocarpa*.**

| **Protein ID** | **Description** | **CGCG *cis*-element and position** |
| --- | --- | --- |
| *Potri.002G100300* | SRS (Seryl-tRNA Synthetase) |  |
| *Potri.005G020200* | CBP60G (Calmodulinbinding protein 60-Like.G) | ACGCGT  (-487 ~ -482)  (-820~-825)  (-1770~1775)  (-1830~-1835) |
| *Potri.013G010700* | CBP60G (Calmodulinbinding protein 60-Like.G) | ACGCGT  (-716~ -721) |
| *Potri.018G019300* | CM2 (Chorismate mutase 2) |  |
| *Potri.012G106000* | ICE1 (Inducer of CBF Expression 1) |  |
| *Potri.015G105200* | ICE1 (Inducer of CBF Expression 1) |  |
| *Potri.004G148300* | XLG2 (Extra-large GTPbinding protein 2) | CCGCGT  (-455 ~ -450) |
| *Potri.009G109400* | XLG2 (Extra-large GTPbinding protein 2) |  |
| *Potri.009G027700* | RHL41/ZAT12 (Responsive to High Light 41) | CCGCGT  (-295 ~ -287)  (-431 ~ -426)  (-1336 ~ -1331) |
| *Potri.001G235800* | RHL41/ZAT12 (Responsive to High Light 41) | CCGCGT  (-815 ~ -890) |
| *Potri.015G136400* | CBF1 (C-Repeat/DRE Binding Factor 1) | CCGCGG  (-1404 ~ -1399) |
| *Potri.012G134100* | CBF1 (C-Repeat/DRE Binding Factor 1) |  |
| *Potri.001G110700* | CBF2 (C-Repeat/DRE Binding Factor2) |  |
| *Potri.015G069600* | EDS1 (Enhanced Disease Susceptibility 1) |  |
| *Potri.012G074700* | EDS1 (Enhanced Disease Susceptibility 1) |  |
| *Potri.012G070000* | EDS16/ ICS1 (Enhanced disease susceptibility to  *Erysiphe orontii*16)/Isochorismate synthase 1 |  |

**Supplementary Table S4.** **Primers for qRT-PCR of *PtCAMTA* genes and alternative splicing of *PtCAMTA*** genes.

| **Gene name** | **Primer (5'-3')** |
| --- | --- |
| *PtCAMTA1* | F GGTGGCACCAGTGAACC |
|  | R TCAGTCAACTAGAGTGAACATC |
| *PtCAMTA1.2* | F GTGGATGGAGCTATTAATGAGGC |
|  | R TCAAATTCCATGTAAAGTGTAAAC |
| *PtCAMTA2* | F ATGGCTGACTCTAGACGTTAC |
|  | R CTGAGCACTTTGCGATCAAAAAG |
| *PtCAMTA2.2* | F GAAGATGCTGAATCAGGATATAATAACC |
|  | R GATGGTAAGTCAAAGACTTTCTGG |
| *PtCAMTA2.3* | F CCATCATGGGAGGATGTATTAG |
|  | R CATACCCTGCCTTTTATCAAAG |
| *PtCAMTA3* | F GTTCTGGAACACAAGCATCACAGA |
|  | R CTTGGTGAATACGAGCAGC |
| *PtCAMTA3.2* | F CAGAAGAAAGATCACAAATAGCCC |
|  | R TCAAGCAACTAGCAAGGAAAAAG |
| *PtCAMTA4* | F GAAGACTGTTAAAGAAGCTCACG |
|  | R GAACCCTCCTGAGTCTCTCGATAG |
| *PtCAMTA4.2* | F CAAAATCAGCCAGAAACAGGCAG |
|  | R TCATTTGTCCACATTCATATCCG |
| *PtCAMTA5* | F TAGCAGCTCTGAACGAAGTG |
|  | R CAATCCAGTTGCACTAACCA |
| *PtCAMTA5.2* | F GTTGAAGCAAAATTTGATCTTGG |
|  | R CAAATTGAAATCAAGCTCCACAGTATC |
| *PtCAMTA5.3* | F CGAATATGATTTTCTGCGAATAAG |
|  | R TCAAGGCTCTGAAGAATAGAGC |
| *PtCAMTA6* | F ATGGAAAGTGGATTCTCGG |
|  | R GATGGTGAAATACTTGTGATTGC |
| *PtCAMTA6.2* | F GGTGAACGTGTTGAGAGATC |
|  | R GATCTCTCAACACGTTCACC |
| *PtCAMTA7* | F GAAACAATCTTGCACCACTTCAGGATG |
|  | R CAAATTCTTGAGGCGGCAAC |
| *PtCAMTA7.2* | F ATGGGTCAAGGATTGACAAATC |
|  | R GCGCCAACTATGACCATCTC |
| *Actin1* | F AGGTTATGCCCTTCCACACG |
|  | R TCGAGGGCAACATACGCAAG |

**Supplementary Table S5. Primers for qRT-PCR of *PuCAMTA*** genes.

| **Gene name** | **Primer (5'-3')** |
| --- | --- |
| *PuCAMTA1* | F ATGCTGCAATCAGGGTAT |
|  | R CTTATTAAATAGAAACAAAG |
| *PuCAMTA2* | F GAAAGCAAACAGAAGA |
|  | R TCAAGAATCTGTAGGCAAGA |
| *PuCAMTA3* | F GAGGATGTATTAGAAAAT |
|  | R CTTTTATCAAAGCTATTG |
| *PuCAMTA4* | F CTATGATAATAGCTGGA |
|  | R ACCTGTATACGCGCTGC |
| *PuCAMTA5* | F GGCTGAGACTAGAAGAT |
|  | R GAGAACCTGCAGGCCTA |
| *PuCAMTA6* | F GGAGCTGCTCGGCCCAG |
|  | R GTCAGGATCATTTGTCAAC |
| *PuCAMTA7* | F CATGCATGCATATGAGGG |
|  | R GACTGGTACATTATCTC |
